# Epidemiological analysis of hospitalized patients at the Clinic for Psychiatry KCV in Novi Sad during 2020

**DOI:** 10.1192/j.eurpsy.2022.1294

**Published:** 2022-09-01

**Authors:** D. Kuljancic

**Affiliations:** University of Novi Sad Medical faculty, Psichiatry, Novi Sad, Serbia

**Keywords:** Epidemiology, Psychiatry, Covid 19

## Abstract

**Introduction:**

The 2020 year was the first year of Covid19 pandemy in Serbia. Epidemiological measures introduced to prevent the spread of the infection have shaped both the everyday life of citizens and the way the health system of our country functions. A large number of those infected required the redistribution of health personnel to work in covid zones and therefore the work with non covid patients suffered.

**Objectives:**

The aim of the study is to process and present the epidemiological characteristics of hospitalized patients at the Clinic for Psychiatry of the Clinical Center of Vojvodina in Novi Sad in 2020.

**Methods:**

A retrospective analytical study of the epidemiological type was conducted.

**Results:**

During 2020, a total of 1345 patients were hospitalized at our Clinic, which is over 30% less than during the previous year. Several males, aged 19 to 45, with a predominant diagnosis of psychosis, were hospitalized. Hospitalizations lasted significantly shorter than during the previous year. The number of relapses was significantly lower. Patients with other diagnoses of mental disorders are significantly less often hospitalized, except for those with addiction diseases who are hospitalized in a reduced percentage.

**Conclusions:**

Restrictive epidemiological measures led to a significant reduction in the number of hospitalizations at our Clinic, primarily because patients were prevented from exercising their right to health care, but also because of the mobilization of all healthy defense mechanisms in a collective crisis situation and consequently reduced psychopathological manifestations.

**Disclosure:**

No significant relationships.

